# Anterior chamber and angle characteristics in Chinese children (6–11 years old) with different refractive status using swept-source optical coherence tomography

**DOI:** 10.1186/s12886-024-03520-8

**Published:** 2024-06-17

**Authors:** Li Li Zhao, Xiu Zhen Lu, Guo Dong Tang, Xiu Yan Zhang, Run Kuan Li, Jing Xu, Jiao Jiao Feng, Zhe Xu, Ji Ke Song, Hong Sheng Bi

**Affiliations:** 1 Shandong University of Traditional Chinese Medicine, Jinan, 250014 PR China; 2 Affiliated Eye Hospital of Shandong University of Traditional Chinese Medicine, Jinan, 250000 PR China; 3 Shandong Provincial Key Laboratory of Integrated Traditional Chinese and Western Medicine for Prevention and Therapy of Ocular Diseases, Key Laboratory of Integrated Traditional Chinese and Western Medicine for Prevention and Therapy of Ocular Diseases in Universities of Shandong, Eye Institute of Shandong University of Traditional Chinese Medicine, Jinan, 250000 PR China

**Keywords:** Swept-source optical coherence tomography, Angle-opening distance, Anterior chamber depth, Axial length, Children

## Abstract

**Background:**

The anatomic structure of the anterior chamber (AC) helps to explain differences in refractive status in school-aged children and is closely associated with primary angle closure (PAC). The aim of this study was to quantify and analyze the anterior chamber and angle (ACA) characteristics in Chinese children with different refractive status by swept-source optical coherence tomography (SS-OCT).

**Methods:**

In a cross-sectional observational study, 383 children from two primary schools in Shandong Province, China, underwent a complete ophthalmic examination. First, the anterior chamber depth (ACD), anterior chamber width (ACW), angle-opening distance (AOD), and trabecular-iris space area (TISA) were evaluated automatically using a CASIA2 imaging device. AOD and TISA were measured at 500, 750 μm nasal (N1 and N2, respectively), and temporal (T1 and T2, respectively) to the scleral spur (SS). Cycloplegic refraction and axial length (AL) were then measured. According to spherical equivalent refraction (SER), the children were assigned to hyperopic (SER > 0.50D), emmetropic (-0.50D < SER ≤ 0.50D), and myopic groups (SER ≤ -0.50D).

**Results:**

Out of the 383 children, 349 healthy children (160 girls) with a mean age of 8.23 ± 1.06 years (range: 6–11 years) were included. The mean SER and AL were − 0.10 ± 1.57D and 23.44 ± 0.95 mm, respectively. The mean ACD and ACW were 3.17 ± 0.24 mm and 11.69 ± 0.43 mm. The mean AOD were 0.72 ± 0.25, 0.63 ± 0.22 mm at N1, T1, and 0.98 ± 0.30, 0.84 ± 0.27 mm at N2, T2. The mean TISA were 0.24 ± 0.09, 0.22 ± 0.09mm^2^ at N1, T1, and 0.46 ± 0.16, 0.40 ± 0.14mm^2^ at N2, T2. The myopic group had the deepest AC and the widest angle. Compared with boys, girls had shorter AL, shallower ACD, narrower ACW, and ACA (all *p <* 0.05). By Pearson’s correlation analysis, SER was negatively associated with ACD, AOD, and TISA. AL was positively associated with ACD, ACW, AOD, and TISA. In the multiple regression analysis, AOD and TISA were associated with deeper ACD, narrower ACW, and longer AL.

**Conclusion:**

In primary school students, the myopic eyes have deeper AC and wider angle. ACD, ACW, AOD, and TISA all increase with axial elongation. ACA is highly correlated with deeper ACD.

**Supplementary Information:**

The online version contains supplementary material available at 10.1186/s12886-024-03520-8.

## Background

The development of ocular structures and refractive status undergo dynamic changes in stages since birth [1]. Children born with mild hyperopia largely complete emmetropization in early childhood and maintain stable refraction during the following years through coordination among ocular components [2]. However, multiple environmental, genetic, and behavioral factors can prevent normal visual development and lead to refractive errors (RE) [3–6]. This is one of the leading causes of vision impairment in schoolchildren [7–9]. Especially after the age of 6 years, there is a clear tendency to myopia [10]. Myopia has become a global concern in recent years, especially in East and Southeast Asian countries [11]. The study of Wang et al. showed that the annual incidence of new-onset myopia among Chinese students starting in grade 1 was 20–30% [12]. The earlier myopia occurs, the easier it is to develop into high myopia in the future, causing more severe visual impairment and disability [13]. As a part of the anterior segment structure, understanding the difference of anterior chamber (AC) in eyes with different refractive status is helpful to identify myopia or pre-myopia in children and conduct early intervention [14, 15]. Several studies showed that the onset and development of myopia are often accompanied by the deepening of anterior chamber depth (ACD) [16] and the widening of anterior chamber angle (ACA) [17, 18]. Further, Chen et al. reported that eyes with more severe myopia tend to have a longer axial length (AL) and a shallower ACD in highly myopic eyes [19].

Primary angle closure glaucoma (PACG) in children is often difficult to be detected in the early stage, and severe damage to the optic nerve may lead to blindness after progression. Therefore, it is necessary to clear the relevant anatomical features and conduct early monitoring in high-risk children [20–23]. Xu et al. found that shallow ACD and narrow ACA were associated with age, hyperopia, female gender, and the presence of PACG [24]. Myopia and long AL are always thought to protect against PACG. However, recent studies found a considerable number of PACG patients with myopia in East Asia [18]. The influence of refractive status and AL on AC and ACA remains unclear.

Currently, techniques used to evaluate AC and angle include gonioscopy, ultrasound biomicroscopy (UBM), optical biometry, Scheimpflug, and OCT imaging [25–27]. However, gonioscopy is a subjective examination, and its inevitable light exposure and pressure on the cornea can cause ACA deformation and widening [28, 29]. Direct contact with UBM reduces patient compliance, particularly in children [30]. Lenstar and Pentacam [31–33] are time-consuming and cannot perform detailed imaging of ACA. Therefore, anterior segment OCT (AS-OCT) has the advantages of high resolution, high accuracy, non-contact imaging, and good repeatability and reproducibility, which can be better applied to the clinical evaluation of AC and angle [34–36]. However, few studies imaged the AC in children using AS-OCT and analyzed its correlation with refraction [37, 38].

Compared to spectral domain OCT (SD-OCT), SS-OCT achieves greater penetration, allowing better observation of tissue structure. In addition, it significantly improves the image acquisition speed, and further improves the axial resolution, scanning range and depth [39, 40]. Thus, our study aimed to investigate the differences in AC and angle characteristics among hyperopic, emmetropic, and myopic children using SS-OCT.

## Methods

### Subjects

This observational, school-based cross-sectional study was conducted from December 2021 to November 2022. 349 children from two primary schools (The Second Primary School of Huantai County and The Fourth Primary School of Huantai County) in Shandong Province, China were included. Detailed demographic information was recorded in the form of a questionnaire survey [41]. Height and weight were measured and BMI was calculated as weight divided by height squared (kg/m^2^).

The protocol for this study was approved by the Ethics Committee of the Affiliated Eye Hospital of Shandong University of Traditional Chinese Medicine (HEC-KS-2020016KY02). The clinical Trial Number is ChiCTR2000039783. Informed consent for cycloplegia and ocular examination was obtained from students who met the inclusion criteria and their parents or legal guardians. This study was conducted in line with the guidelines of the Declaration of Helsinki. Subjects were selected using a two-stage random sampling method. Two of the five primary schools in Huantai County were first randomly selected, and then an equal proportion of children were randomly selected from each grade in the two primary schools.

Inclusion criteria were the following:

1. Students in grades 1 to 5 (aged 6–11 years);
2. 20/25 or better best-corrected visual acuity (BCVA);
3. IOP ≤ 21 mm Hg.

Exclusion criteria were as follows:

1. Having glaucoma, strabismus, amblyopia, other ocular diseases, or systemic diseases;
2. History of intraocular surgery or ocular trauma;
3. Contact lens wearers;
4. Could not cooperate during SS-OCT measurements.

### Ophthalmic examinations

Each participant underwent a comprehensive ophthalmic examination including BCVA, slit-lamp microscope, intraocular pressure (IOP), and cycloplegic refractometry.

1% cyclopentolate eye drops (Alcon, Ft. Worth, Texas, USA), a cycloplegic agent, were applied 3 times with an interval of 5 min. At least 20 min after the last eye drop, when the pupillary light reflex disappeared, autorefraction was performed with an auto-refractor (ARK-1, NIDEK, JAPAN) [25]. The mean of three measurements was used to determine the SER, which was calculated as the spherical refractive power plus half of the cylindrical refractive power.

AL and IOP were measured by a laser interferometry (IOL-Master, V5.0, Carl Zeiss Meditec AG, Jena, Germany) and a non-contact tonometer (NT-510, NIDEK, Japan), respectively. Given the high correlation between the two eyes, only data from the right eye were analyzed. According to refractions, the subjects were assigned to the hyperopic (SER > 0.50D), emmetropic (-0.50D < SER ≤ 0.50D), and myopic groups (SER ≤ -0.50D).

### SS-OCT examination

All subjects underwent anterior segment imaging using a CASIA2 SS-OCT (Tomey Corp, Nagoya, Japan) in a standard dark condition (< 5 lx) prior to cycloplegic instillation. CASIA2 is a second-generation anterior segment OCT using the principle of low coherent reflection, with a wavelength of 1310 nm. The further optimization of scanning speed (5, 0000 scans/s), measurement range (16 × 13 mm), density (13 mm), and imaging resolution (10 μm axial, 30 μm transverse) enables a continuous series of clear images to be obtained, and the relevant parameters were automatically calculated using the built-in analysis software (Version 3E.22).

**Fig. 1:**
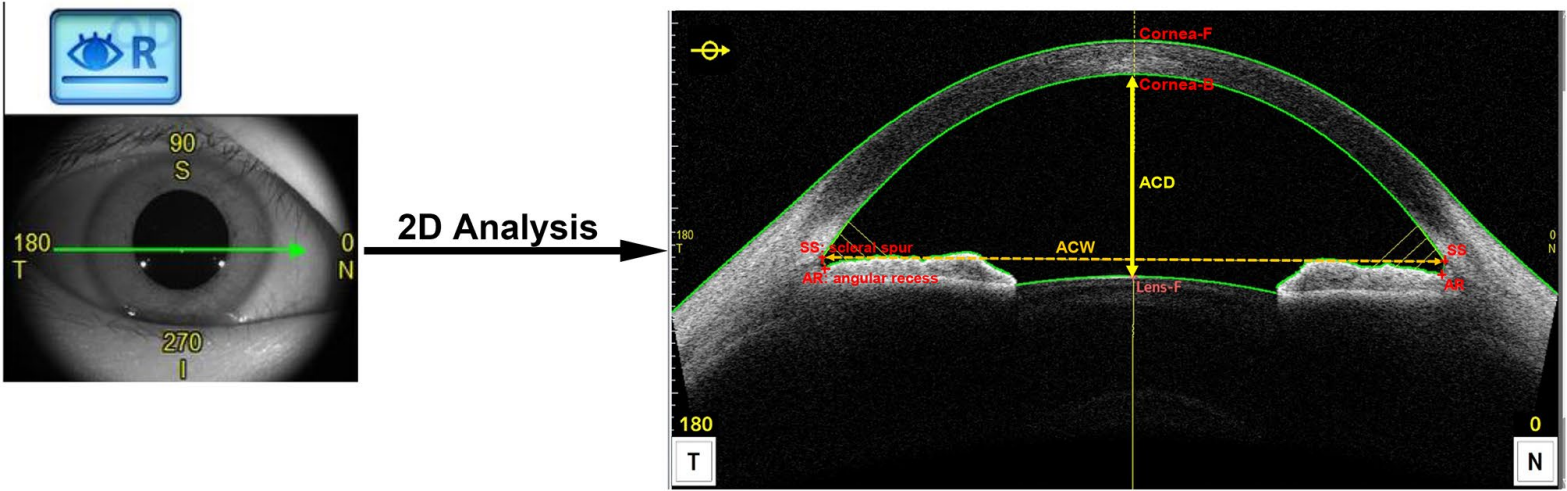
CASIA2 used the “2D Analysis” module to calculate anterior chamber depth (ACD) and anterior chamber width (ACW). The green arrow indicated the scanning direction. ACD was defined as the axial distance from the posterior surface of the cornea to the anterior surface of the lens. ACW was defined as the distance between the nasal and temporal scleral spur

The Lens Biometry mode was used for AC imaging, and the scanning protocol consisted of continuous meridional scanning (800 A-scans per row). Sixteen different 2D images can be generated. The “2D Analysis” module was used for automatic identification and labeling, including nasal and temporal scleral spur (SS), angular recess (AR), anterior and posterior corneal surfaces, and anterior lens surfaces. The SS as an initial label was identified first along the interface between the hyporeflective ciliary muscle and the hyperreflective sclera. And then the anterior and posterior boundaries of the cornea and iris were automatically segmented (See Fig. 1).

**Fig. 2:**
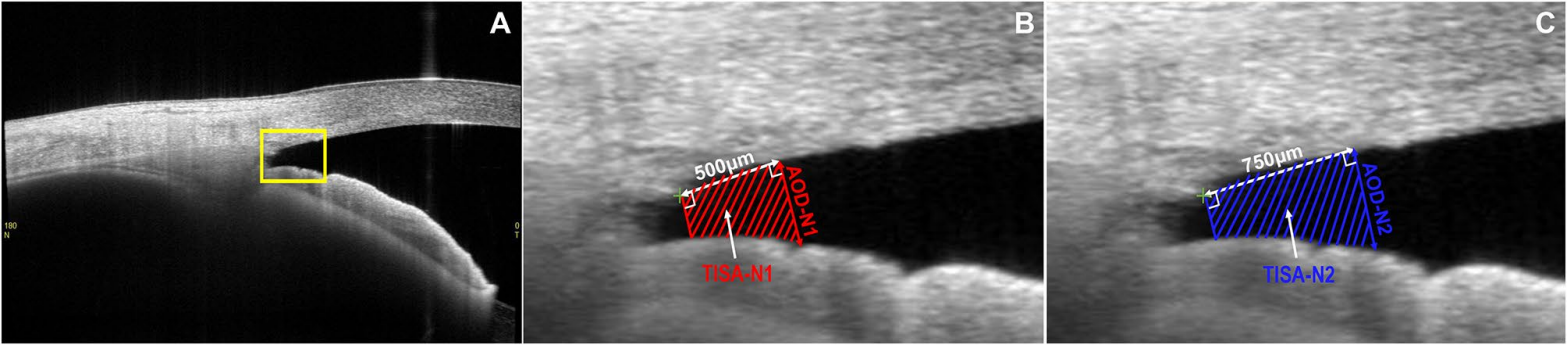
(**A**) The original image annotated with the angle parameters in nasal quadrant (magnify the area inside the yellow box). (**B, C** N1 and N2 are located 500 μm and 750 μm, respectively, to the SS in the nasal quadrant. T1 and T2 are located 500 μm and 750 μm, respectively, to the SS in the temporal quadrant. AOD-N1/N2 is the vertical distance from the trabecular meshwork at 500/750µm anterior to the SS, to the anterior surface of the iris. TISA-N1/N2 is the shaded area with the following boundaries: anterior, AOD-N1/N2; posteriorly, a line drawn from the SS perpendicular to the plane of the inner scleral wall to the opposing iris; superior, the inner corneoscleral wall; and inferior, the iris surface

ACA images were acquired using a 3D angle analysis scanning protocol. Each volume consisted of 128 radial B-scans, each 16 mm in length and 6 mm in depth. AOD and TISA (Fig. 2B and C) in the nasal and temporal quadrants were analyzed using the 360° SS-OCT viewer software (V.6.0, Tomey, Nagoya, Japan). The selected images showed good SS and AR. SS was confirmed or fine-tuned based on automated markers by the same experienced observer who was blinded to clinical data. Low-quality images with severe motion artifacts were excluded.

### Statistical analysis

All statistical analyses were performed by SPSS 21.0 software (IBM SPSS Statistics, New York, NY, USA). Kolmogorov-Smirnov test was used to verify the normality of variables and to determine the subsequent statistical methods. Continuous variables were expressed as mean ± standard deviation (SD). Comparisons of different refractive groups were performed by the one-way analysis of variance (ANOVA) with Bonferroni adjustment for multiple comparisons. Independent sample t-test was used to compare the differences in each parameter between genders and in nasal and temporal angles. Pearson correlation analysis and simple linear regression analysis were used to evaluate the relationship between AC (ACD, ACW), ACA (AOD, TISA) and SER, AL. The factors that could best explain the angle parameters were determined by the stepwise forward multiple linear regression analysis. All *p* values were from 2-sided tests and *p* < 0.05 was considered statistically significant.

## Results

### Subject characteristics

After excluding 34 eyes that did not meet the inclusion criteria (4 eyes had IOP > 21 mmHg, 5 eyes had strabismus or amblyopia, 4 eyes wearing corneal contact lens) or had low image quality (21 eyes), 349 (91.1%) children’s right eyes were included in the final analysis. The mean age was 8.23 ± 1.06 years old (range, 6 to 11 years old) and 160 were girls (45.8%). The demographics were depicted in Table 1. Overall, the mean SER and AL were − 0.10 ± 1.57D (range, -6.50 to 3.875 D) and 23.44 ± 0.95 mm (range, 21.52 to 26.51 mm), respectively.

**Table 1:** The demographic characteristics of the participants

Parameters	Boys (*n* = 189)			Girls (*n* = 160)			Total (*n* = 349)	*P*(age)	*P*(sex)
	6-7y	8-9y	10-11y	6-7y	8-9y	10-11y			
BMI (kg/m^2^)	17.11 ± 2.70	18.71 ± 3.66	20.60 ± 4.31	16.67 ± 3.10	17.43 ± 3.50	19.85 ± 4.32	18.10 ± 3.70	< 0.001	0.004
IOP (mmHg)	16.25 ± 2.90	16.91 ± 2.86	16.87 ± 2.54	16.92 ± 2.81	17.02 ± 2.90	17.05 ± 2.12	16.86 ± 2.80	0.575	0.417
SER (D)	0.40 ± 0.79	0.06 ± 1.47	-1.35 ± 1.93	0.72 ± 0.78	-0.35 ± 1.75	-1.02 ± 1.76	-0.10 ± 1.57	< 0.001	0.898
AL (mm)	23.30 ± 0.60	23.67 ± 0.91	24.36 ± 1.09	22.67 ± 0.66	23.23 ± 0.89	23.84 ± 0.77	23.44 ± 0.95	< 0.001	< 0.001

Values are presented as mean ± SD

Gender differences were compared using independent samples t-test

Age differences were compared using ANOVA

Table 2 summarized the AC and ACA measurements. The mean ACD and ACW were 3.17 ± 0.24 mm and 11.69 ± 0.43 mm. By independent samples t-test, the AOD were 0.72 ± 0.25 mm, 0.63 ± 0.22 mm at N1, T1 (*p* < 0.001), and 0.98 ± 0.30 mm, 0.84 ± 0.27 mm at N2, T2 (*p* < 0.001). The TISA were 0.24 ± 0.09 mm^2^, 0.22 ± 0.09 mm^2^ at N1, T1 (*p* < 0.001), and 0.46 ± 0.16 mm^2^, 0.40 ± 0.14 mm^2^ at N2, T2 (*p* < 0.001). Among the three age groups, participants aged 10–11 had the longest AL, the deepest ACD, and the widest ACA (all *p* < 0.001). All parameters except SER and IOP revealed significant gender differences. Girls have shorter AL, shallower AC, and narrower ACA than boys (all *p* < 0.05).

**Table 2:** The AC and ACA characteristics of the participants

Parameters			Boys			Girls			Total	P(age)	P(sex)
			**6-7y**	**8-9y**	**10-11y**	**6-7y**	**8-9y**	**10-11y**			
AC	**ACD (mm)**		3.14 ± 0.14	3.24 ± 0.23	3.27 ± 0.23	3.04 ± 0.21	3.11 ± 0.26	3.20 ± 0.33	3.17 ± 0.24	< 0.001	< 0.001
	**ACW (mm)**		11.68 ± 0.36	11.75 ± 0.41	11.80 ± 0.43	11.67 ± 0.50	11.58 ± 0.42	11.87 ± 0.37	11.69 ± 0.43	0.068	0.022
ACA	**AOD (mm)**	**N1**	0.65 ± 0.16	*0.81 ± 0.27*	0.78 ± 0.24	0.62 ± 0.16	0.68 ± 0.24	0.68 ± 0.32	0.72 ± 0.25	< 0.001	< 0.001
		**N2**	0.90 ± 0.21	1.10 ± 0.32	1.06 ± 0.30	0.92 ± 0.29	0.90 ± 0.21	0.92 ± 0.37	0.98 ± 0.30	< 0.001	< 0.001
		**T1**	0.59 ± 0.17	0.69 ± 0.25	0.69 ± 0.22	0.56 ± 0.16	0.58 ± 0.21	0.57 ± 0.27	0.63 ± 0.22	0.030	< 0.001
		**T2**	0.80 ± 0.20	0.93 ± 0.28	0.93 ± 0.27	0.74 ± 0.19	0.79 ± 0.26	0.75 ± 0.32	0.84 ± 0.27	0.014	< 0.001
	**TISA (mm** ^**2**^ **)**	**N1**	0.22 ± 0.05	0.28 ± 0.10	0.26 ± 0.09	0.21 ± 0.07	0.23 ± 0.09	0.23 ± 0.11	0.24 ± 0.09	0.001	< 0.001
		**N2**	0.41 ± 0.10	0.52 ± 0.18	0.49 ± 0.15	0.39 ± 0.11	0.43 ± 0.15	0.44 ± 0.20	0.46 ± 0.16	< 0.001	< 0.001
		**T1**	0.20 ± 0.06	0.24 ± 0.10	0.26 ± 0.08	0.19 ± 0.06	0.20 ± 0.08	0.20 ± 0.10	0.22 ± 0.09	0.012	< 0.001
		**T2**	0.38 ± 0.10	0.44 ± 0.16	0.45 ± 0.14	0.35 ± 0.10	0.38 ± 0.14	0.37 ± 0.18	0.40 ± 0.14	0.015	< 0.001

Values are presented as mean ± SD

Gender differences were compared using independent samples t-test

Age differences were compared using ANOVA

### AC and ACA parameters in different groups

**Fig. 3:**
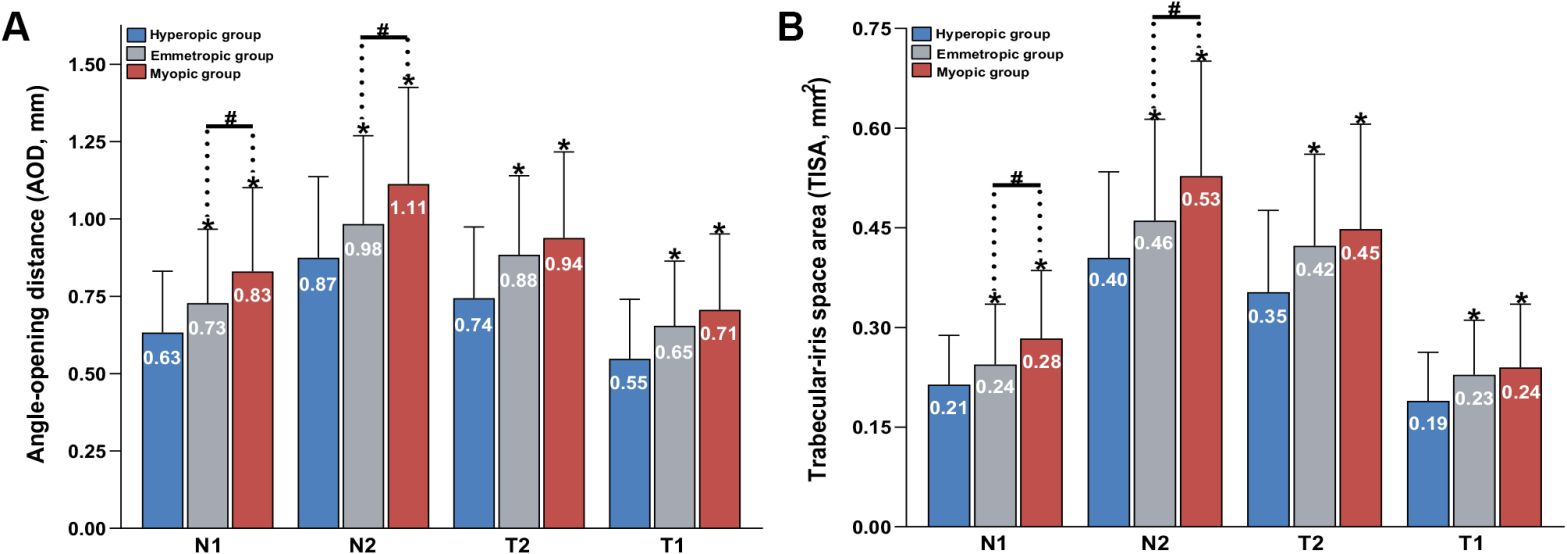
Comparison of ACA parameters in the horizontal quadrant of subjects according to different refractive status N1, 500 μm to the scleral spur (SS) in nasal quadrant; N2, 750 μm to the SS in nasal quadrant; T1, 500 μm to the SS in temporal quadrant; T2, 750 μm to the SS in temporal quadrant. * indicates a significant difference between the emmetropia/myopic group and the hyperopic group (*p* < 0.05). # indicates a significant difference between the myopic and emmetropic groups (*p* < 0.05)

Table 3 shows the differences in ACD, ACW, AOD, and TISA among different SER groups. The myopic group presented the deepest ACD (F = 25.53, *P* < 0.001). However, there was no significant difference in ACW among the three groups (F = 0.996, *P* = 0.371). Figure 3 shows that AOD and TISA increased sequentially at all locations in the hyperopic, emmetropic, and myopic groups.

**Table 3:** Comparison of AC and ACA parameters in subjects according to different refractive status

Variables			Hyperopia (*n* = 146)	Emmetropia (*n* = 98)	Myopia (*n* = 98)	F-value	P-value*			
							*Overall*	*Hyperopia vs. Emmetropia*	*Hyperopia vs. Myopia*	*Emmetropia vs. Myopia*
AC	**ACD (mm)**		3.07 ± 0.23	3.20 ± 0.24	3.27 ± 0.21	25.53	< 0.001	< 0.001	< 0.001	0.083
	**ACW (mm)**		11.71 ± 0.45	11.72 ± 0.46	11.65 ± 0.36	0.996	0.371	1.000	0.699	0.615
ACA	**AOD (mm)**	**N1**	0.63 ± 0.20	0.73 ± 0.24	0.83 ± 0.27	21.93	< 0.001	0.006	< 0.001	0.006
		**N2**	0.87 ± 0.260	0.98 ± 0.29	1.11 ± 0.31	21.41	< 0.001	0.027	< 0.001	0.005
		**T1**	0.55 ± 0.19	0.65 ± 0.21	0.71 ± 0.25	17.97	< 0.001	< 0.001	< 0.001	0.255
		**T2**	0.74 ± 0.23	0.88 ± 0.26	0.94 ± 0.28	19.93	< 0.001	0.001	< 0.001	0.993
	**TISA (mm** ^**2**^ **)**	**N1**	0.21 ± 0.07	0.24 ± 0.09	0.28 ± 0.10	18.82	< 0.001	0.010	< 0.001	0.004
		**N2**	0.40 ± 0.13	0.46 ± 0.15	0.53 ± 0.17	20.55	< 0.001	0.014	< 0.001	0.005
		**T1**	0.19 ± 0.07	0.23 ± 0.08	0.24 ± 0.10	12.99	< 0.001	< 0.001	< 0.001	0.377
		**T2**	0.35 ± 0.12	0.42 ± 0.14	0.45 ± 0.16	15.99	< 0.001	< 0.001	< 0.001	0.598

Values are presented as mean ± SD

*One-way ANOVA with post Bonferroni test

ACD: anterior chamber depth; ACW: anterior chamber width; AOD-N1/N2: angle opening distance measured at 500–750 μm from the scleral spur in the nasal quadrant; AOD-T1/T2: angle opening distance measured at 500–750 μm from the scleral spur in the temporal quadrant; TISA-N1/N2: trabecular-iris space area measured at 500–750 μm from the scleral spur in the nasal quadrant; TISA-T1/T2: trabecular-iris space area measured at 500–750 μm from the scleral spur in the temporal quadrant

**Fig. 4:**
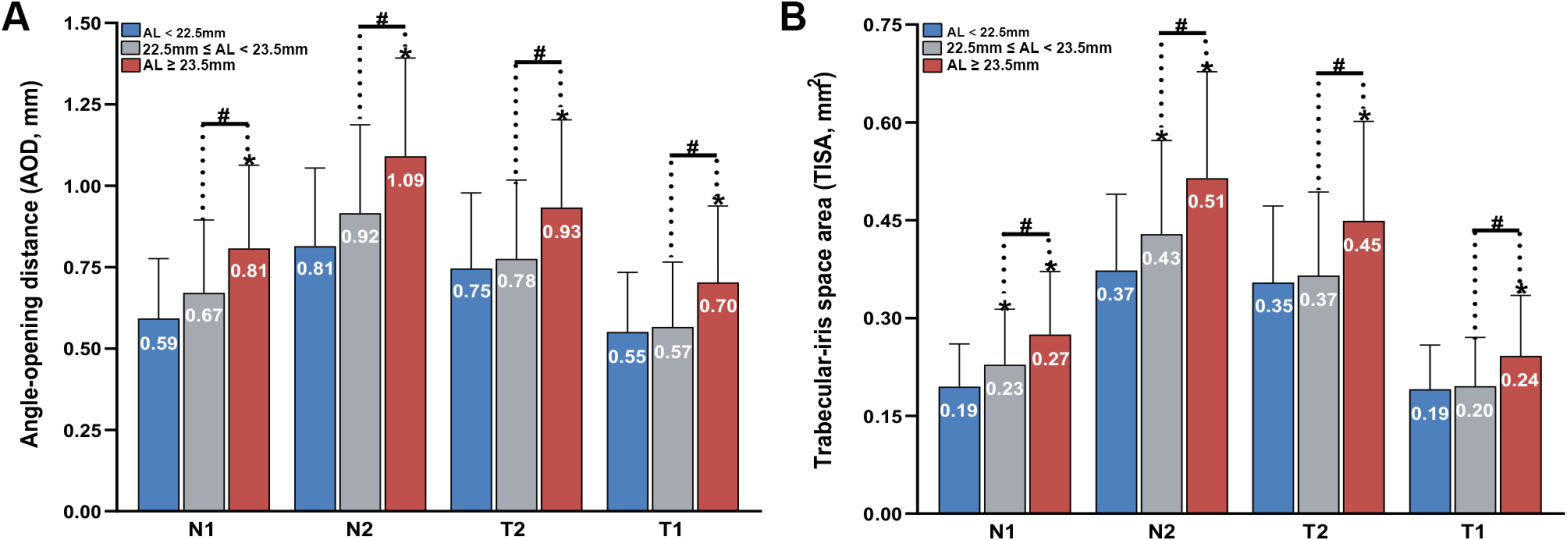
Comparison of ACA parameters in the horizontal quadrant of subjects according to different AL. N1, 500 μm to the scleral spur (SS) in nasal quadrant; N2, 750 μm to the SS in nasal quadrant; T1, 500 μm to the SS in temporal quadrant; T2, 750 μm to the SS in temporal quadrant. * indicates a significant difference between the eyes with 22.5 mm ≤ AL < 23.5 mm /AL ≥ 23.5 mm and eyes with AL < 22.5 mm. # indicates a significant difference between eyes with 22.5 mm ≤ AL < 23.5 mm and AL ≥ 23.5 mm

We further compared the AC and ACA parameters in eyes with different axial lengths. Table 4 presents the eyes with the longest AL had the deepest ACD (F = 53.87, *P* < 0.001), the widest ACW (F = 32.63, *p* < 0.001), the largest AOD and TISA (all *P* < 0.001). Figure 4 presents that the nasal angle width grew more significantly with AL than the temporal angle.

**Table 4:** Comparison of AC and ACA parameters in subjects with different axial length

Variables			AL < 22.5 mm (*n* = 62)	22.5 mm ≤ AL < 23.5 mm (*n* = 129)		AL ≥ 23.5 mm (*n* = 158)	F-value	P-value*				
								*Overall*	*AL < 22.5 mm vs. 22.5 mm ≤ AL < 23.5 mm*	*22.5 mm ≤ AL < 23.5 mm vs. AL ≥ 23.5 mm*	*AL < 22.5 mm vs. AL ≥ 23.5 mm*	
AC	**ACD (mm)**		2.97 ± 0.23	3.11 ± 0.21	3.29 ± 0.20		58.58	< 0.001	< 0.001	< 0.001	< 0.001	
	**ACW (mm)**		11.35 ± 0.35	11.67 ± 0.41	11.86 ± 0.39		39.15	< 0.001	< 0.001	< 0.001	< 0.001	
ACA	**AOD (mm)**	**N1**	0.59 ± 0.18	0.67 ± 0.22	0.81 ± 0.26		23.52	< 0.001	0.088	< 0.001	< 0.001	
		**N2**	0.81 ± 0.24	0.92 ± 0.27	1.09 ± 0.30		26.41	< 0.001	0.062	< 0.001	< 0.001	
		**T1**	0.55 ± 0.18	0.57 ± 0.20	0.70 ± 0.23		19.15	< 0.001	1.000	< 0.001	< 0.001	
		**T2**	0.75 ± 0.23	0.78 ± 0.24	0.93 ± 0.27		19.22	< 0.001	1.000	< 0.001	< 0.001	
	**TISA (mm** ^**2**^ **)**	**N1**	0.19 ± 0.07	0.23 ± 0.09	0.27 ± 0.10		20.46	< 0.001	0.041	< 0.001	< 0.001	
		**N2**	0.37 ± 0.12	0.43 ± 0.14	0.51 ± 0.16		23.99	< 0.001	0.047	< 0.001	< 0.001	
		**T1**	0.19 ± 0.07	0.20 ± 0.07	0.24 ± 0.09		14.76	< 0.001	1.000	< 0.001	< 0.001	
		**T2**	0.35 ± 0.12	0.37 ± 0.13	0.45 ± 0.15		17.31	< 0.001	1.000	< 0.001	< 0.001	

Values are presented as mean ± SD

*One-way ANOVA with post Bonferroni test

ACD: anterior chamber depth; ACW: anterior chamber width; AOD-N1/N2: angle opening distance measured at 500–750 μm from the scleral spur in the nasal quadrant; AOD-T1/T2: angle opening distance measured at 500–750 μm from the scleral spur in the temporal quadrant; TISA-N1/N2: trabecular-iris space area measured at 500–750 μm from the scleral spur in the nasal quadrant; TISA-T1/T2: trabecular-iris space area measured at 500–750 μm from the scleral spur in the temporal quadrant

### Associations between AC (ACD, ACW) and SER, AL

**Fig. 5:**
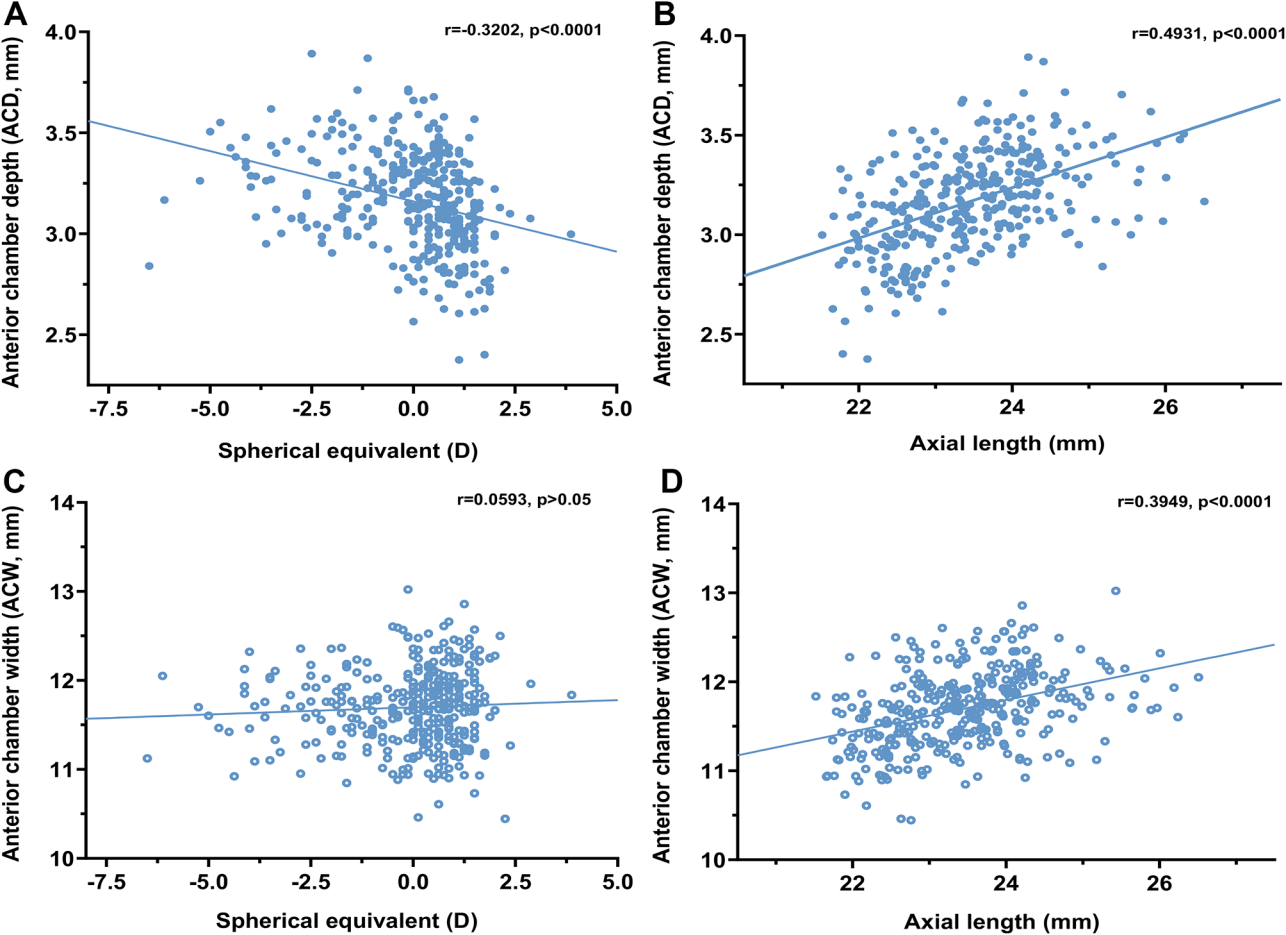
Relationship between the ACD, ACW and SER, AL. (**A**) SER was negatively correlated with ACD (*r* = -0.320, *p* < 0.0001). (**B**) AL was positively correlated with ACD (*r* = 0.493, *p* < 0.0001). (**C**) SER was not significantly correlated with ACW (*r* = 0.059, *p* > 0.05). (**D**) AL was positively correlated with ACW (*r* = 0.395, *p* < 0.0001)

Figure 5 depicts the relationship between ACD, ACW and SER, AL. SER was found to be negatively correlated with ACD (*r* = 0.32, *p* < 0.0001, Fig. 5A), but not with ACW (*r* = 0.06, *p* = 0.004, Fig. 5C). AL had a significant positive correlation with ACD (*r* = 0.49, *p* < 0.0001, Fig. 5B) and ACW (*r* = 0.39, *p* < 0.0001, Fig. 5D). A larger AL implied a deeper and wider AC.

### Associations between ACA (AOD, TISA) and SER, AL

**Fig. 6:**
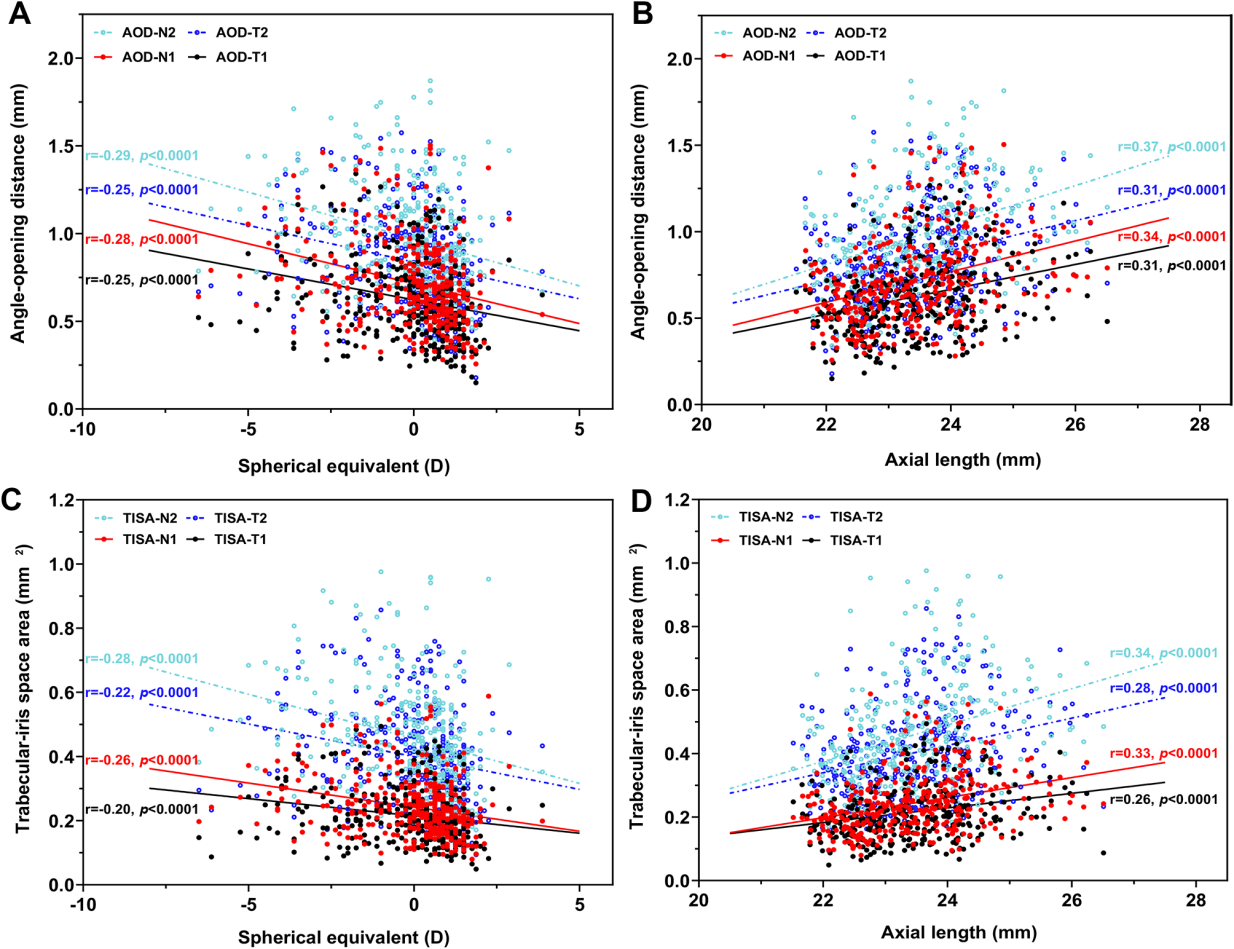
Relationship between angle-opening distance (AOD_500//750_), trabecular-iris space area (TISA_500//750_), and SER, AL in nasal and temporal quadrants. (**A**) SER showed a significant negative effect of AOD_500_ in nasal (*r* = -0.28, *p* < 0.001) and temporal (*r* = -0.25, *p <* 0.001) quadrants. There was also a significant negative correlation between SER and AOD_750_ in nasal (*r* = -0.29, *p <* 0.001) and temporal (*r* = -0.25, *p <* 0.001) quadrants. (**B**) AL was positively correlated with AOD_500_ in nasal (*r* = 0.34, *p <* 0.001) and temporal (*r* = 0.31, *p <* 0.001) quadrants. AL was also strongly positive associated with AOD_750_ in nasal (*r* = 0.37, *p <* 0.001) and temporal (*r* = 0.31, *p <* 0.001) quadrants. (**C**) SER significantly positively correlated with nasal TISA_500_ (*r* = -0.26, *p <* 0.001), temporal TISA_500_ (*r* = -0.20, *p <* 0.001), nasal TISA_750_ (*r* = -0.28, *p <* 0.001), and temporal TISA_750_ (*r* = -0.22, *p <* 0.001). (**D**)AL was positively correlated with TISA_750_ in nasal (*r* = 0.33, *p <* 0.001) and temporal (*r* = 0.26, *p <* 0.001) quadrants. AL was also strongly associated with TISA_750_ in nasal (*r* = 0.34, *p <* 0.001) and temporal (*r* = 0.28, *p <* 0.001) quadrants

Figure 6 demonstrates the correlation between AOD, TISA and SER, AL. SER was revealed to be significantly negatively correlated with AOD and TISA (all *p* < 0.0001, Fig. 6A, C). AL had a strong positive correlation with AOD and TISA (all *p* < 0.0001, Fig. 6B, D). The correlation of SER and AL with nasal angle was stronger than that with temporal angle.

### Multiple linear regression

In multiple linear regression analysis, all variables (age, gender, BMI, SER, AL, IOP, ACD, ACW) were included in the initial model of exploratory testing to gradually determine the best predictors of angle parameters. The final regression model was presented in Table 5. All angle parameters were significantly positively correlated with the deeper ACD and negatively correlated with the wider ACW. In addition, female gender was only significantly associated with AOD-T2 and TISA-T2. ACD was found to explain 30.9% and 36.3% of the variability in AOD-N1 and AOD-N2. It was also found to explain 24.3% and 29.2% of the variability in TISA-N1 and TISA-N2.

**Table 5:** Multiple regression analysis between AOD, TISA and other factors

Parameters		Significant factor	Standardized regression coefficient beta	Variance inflation factor	*P* value	*R* ^2^
AOD (mm)	N1	ACD ACW AL	0.595 -0.245 0.143	1.438 1.310 1.375	< 0.001 < 0.001 0.005	0.361
	N2	ACD ACW AL	0.638 -0.207 0.136	1.438 1.310 1.375	< 0.001 < 0.001 0.006	0.401
	T1	ACD ACW AL	0.553 -0.256 0.131	1.438 1.310 1.375	< 0.001 < 0.001 0.014	0.301
	T2	ACD ACW Gender	0.622 -0.256 0.122	1.294 1.240 1.059	< 0.001 < 0.001 0.007	0.356
TISA (mm^2^)	N1	ACD ACW AL	0.514 -0.221 0.158	1.438 1.310 1.375	< 0.001 < 0.001 0.003	0.289
	N2	ACD ACW AL	0.567 -0.227 0.153	1.438 1.310 1.375	< 0.001 < 0.001 0.004	0.339
	T1	ACD	0.543	1.239	< 0.001	0.238
		ACW	-0.232	1.239	< 0.001	
	T2	ACD ACW Gender	0.567 -0.243 -0.103	1.294 1.240 1.059	< 0.001 < 0.001 0.029	0.292

## Discussion

In recent years, the continual advancement of AS-OCT resolution, tissue penetration, and analytic technology has supplied essential baseline information for understanding the anatomical properties of the anterior segment as well as diagnosing and monitoring relevant diseases [42–44]. The AC and angle characteristics of Chinese children were investigated in this study using the latest SS-OCT. We did OCT measurements before cycloplegia because it can cause ACD to be overestimated [45]. The nasal and temporal angles were selected for analysis because children’s cooperation was low and subjects were required to open their eyes naturally during the measurement to avoid pressure on the eyeballs, so it was inevitable that some superior and inferior angles would be covered. In addition, it has been reported that the nasal and temporal angles have better visibility of the scleral spur compared to the superior and inferior angles [46].

Studies investigating the influence of different ethnic groups on AC and angle measurements revealed that ACD, ACW, AOD, and TISA were all significantly smaller in Chinese than in Whites and Japanese [23, 47]. Our results showed high agreement with OCT measurements in South Asian children [48]. Our mean AL (23.44 mm vs. 23.65 mm) and ACD (3.17 mm vs. 3.22 mm) were generally consistent with the results of a large sample study conducted on Shanghai children [25]. It should be noted that the mean AL and ACD measured by Jin et al. were 23.80 mm and 2.70 mm. The longer AL but shallower ACD suggest that their subjects may have thicker lenses or longer posterior segments [38]. Several previous studies also suggested that increasing lens curvature and lens thickness may contribute to the narrowing of ACA in adults [49–51]. Future research on the mechanism of the correlation between lens parameters and ACA will be helpful for the prediction of PACG.

Consistent with previous findings [33, 52], we found a longer AL and a deeper ACD in myopic eyes (*P* < 0.001). Hosny et al. used UBM to measure ACD in adults and found it was significantly associated with SER (*r* = -0.623, *p <* 0.01) and AL (*r* = 0.531, *p <* 0.01) [53]. In the present study, we found that ACD was negatively correlated with SER (*r* = -0.320, *p* < 0.0001) and positively correlated with AL (*r* = 0.493, *p* < 0.0001), but the correlation was lower than in adults. Terasaki et al. found that total AL elongation was significantly greater within 1 year in eyes with myopic biological features such as a deeper anterior chamber depth, thinner lens thickness, and longer AL. The growth rate change tended to accelerate in the eyes with hyperopic ocular biometry during the 1st year only in girls. Individual differences in AL elongation rate may be influenced by ocular biometry [54]. A previous study reported a weak but significant association between ACW and SER (*r* = 0.10, *p* < 0.05), but their study was conducted in a large sample of children and adults. We noted that in their study, ACW was essentially unchanged at ages 6–8 to 10–14 years, but gradually narrowed in adulthood [31].

Several studies reported the distribution and influencing factors of ACA parameters in children [25, 31, 37], but the results were inconsistent. We found that a narrower ACA was associated with younger age, female sex, hyperopia, shorter AL, and shallower ACD. Similar to our study, Edawaji et al. also found a weak but significant negative correlation between SER and ACA [31]. In addition, Fermendez-Vigo et al. detected a stronger correlation between AOD500 (*r* = -0.545, *p <* 0.001), TISA_500_ (*r* = -0.540, *p <* 0.001) and SER in adults by Fourier-domain OCT [55]. Combined with previous studies in different age ranges on AL, AC, and angle [17, 38, 56–58], we speculate that AC and AL increase is synchronized in early childhood. The ACD deepened with the extension of AL, and the ACA widened with the deepening of ACD. In the Pentacam results of Wang et al., ACA was the narrowest in myopic children, followed by hyperopic children [25]. And they suggested that a long AL might lead to a narrower ACA. In a previous study of Asian angle-closure patients, it was found that about a quarter of them had myopia. Their AL and vitreous length (VL) were significantly longer than other patients, but there was no significant difference in ACD [18]. Therefore, not all myopic eyes have deep ACD, and some axial myopic eyes still show shallow AC and narrow ACA. The AC and angle parameters peaked at a certain period in childhood and then gradually decreased again with age. Myopia may also be accompanied by narrowing or even closure of the angle [17, 25]. In our study, the children were in a period when ACD increased with AL [59].

In the final multiple regression analysis, deeper ACD, narrower ACW, longer AL, and girls were associated with AOD and TISA. ACD can explain most of the variation in AOD and TISA. Jin et al. found that age and ACD explained approximately 50% of the variability in AOD and TISA among healthy Chinese children [38]. Of note, gender was only associated with temporal AOD_750_ and TISA_750_. Most studies have suggested that narrow angles are associated with narrower ACW [60]. However, in our study, wider ACW was associated with narrower angles, which may be explained by the higher proportion of children with longer AL (≥ 23.5 mm) in our subjects. In a study conducted by Li et al., primary angle closure (PAC) patients with longer AL (≥ 23.5 mm) had wider ACW and flatter corneas compared to patients with relatively shorter AL (< 22.5 mm) [61]. This is consistent with Zhang et al., who suggested that lower SE in atypical PAC subjects was attributed to relatively longer AL. In PAC patients, larger ACD and ACW indicate greater vertical and horizontal dimensions in the anterior segment of the eyeball [62].

In our study, we found significant gender differences. This difference persisted after adjustment for age. Compared with boys, girls had shorter AL, shallower ACD, narrower ACW, and ACA (all *p <* 0.05). Nadeem et al. observed gender differences in Pakistani children similar to ours, but not significant [48]. AC measurements by handheld OCT showed that girls had narrower ACW and ACA than boys throughout childhood in UK children [31]. In addition, Hashemi et al. reported that girls had thicker lenses than boys. Since there was no significant gender difference in SER in this study, it can be speculated that boys may have a flatter corneal curvature and a larger posterior lens than girls. However, some investigators believe that gender is not related to ACA structure [37, 38]. There were significant differences in AC and angle among subjects of different ages. In other studies with larger age ranges (3 to 18 years and 7 to 15 years), ACD, AOD, and TISA also showed weak positive associations with age [37, 38]. Interestingly, our children had the same trend as White children [37], with a widening ACA at ages 6–9 years and a narrowing ACA at ages 10–11 years. Another finding supporting previous studies was the significant difference in ACA between the nasal and temporal quadrants (all *p* < 0.001) [49, 63]. But we cannot definitively explain this difference at present. Although Edawaji et al. found no difference in nasal and temporal angle width (*p* > 0.05), they found significant differences in nasal and temporal trabecular network length between ages 5 to 18 (*p* < 0.05) [31].

This study’s strength is the first use of SS-OCT to quantify AC and angle in Chinese children and to investigate their relationships with SER and AL. Limitations were first shown in the narrow age group. The conclusions in this study cannot be generalized to other ages. Second, we did not quantify the complete angle because it was difficult for the children to cooperate. In addition, other AC parameters (iris thickness, iris curvature, AC area, AC volume) were not measured due to the limitation of the analytical model. In the future, we may undertake longitudinal cohort studies in the same children to explore the dynamic association of the development of AC and angle with age and refractive change. We also considered evaluating AC and angle in children with glaucoma or high myopia for comparison with normal children.

## Conclusions

In summary, there were significant differences in AC and angle parameters among children with different refractive status. They changed synchronously with AL extension. Shallow ACD is the main cause for the narrow ACA. The use of AS-OCT technology in children may be helpful for the initial screening and diagnosis of anterior segment disorders.

### Electronic supplementary material

Below is the link to the electronic supplementary material.

Supplementary Material 1

## Data Availability

The data generated or analyzed to support the findings of this study are available from the corresponding author on reasonable request.
