# Supplementary material for: Anterior chamber and angle characteristics in Chinese children (6–11 years old) with different refractive status using swept-source optical coherence tomography
Source: BMC Ophthalmol. 2024 Jun 17;24:259. doi: 10.1186/s12886-024-03520-8 (PMC11181599; doi:10.1186/s12886-024-03520-8)
Supplement: Supplementary file 1 — Supplementary Material 1 [file 12886_2024_3520_MOESM1_ESM.pdf]

Epidemiological investigation of basic information

Detailed information

Name:

Gender:

Nationality:

School:

Grade:

Class:

Questionnaire survey

1. Whether the child is myopic?

A. Yes

B. No

2. Choose the type of glasses your child is currently wearing.

A. Frame glasses

B. Contact lens

C. Orthokeratology lenses

D. Don't wear glasses

E. Other

3. When did you first discover your child's myopia?

4. How to discover the child myopia at that time?

A. Blurred distance vision

B. Through the school physical examination

C. It was found during a hospital examination

D. It was found during the optician's inspection

E. Other
